# Effects of a low, medium, and high-intensity aquatic physiotherapy protocol on functional and biochemical parameters in individuals with knee osteoarthritis: protocol for a crossover randomized controlled trial

**DOI:** 10.12688/f1000research.140342.3

**Published:** 2024-06-24

**Authors:** Cesar Augusto Teixeira, Lisete Haas, Bruna Frata, Andressa Fiori Bortoli, Fernanda Scalco Acco, Gustavo de Castro, Fernanda Cechetti

**Affiliations:** 1 Graduate Program in Rehabilitation Sciences, Universidade Federal de Ciencias da Saude de Porto Alegre, Porto Alegre, State of Rio Grande do Sul, Brazil; 2 Department of Physiotherapy, Universidade FEEVALE, Novo Hamburgo, State of Rio Grande do Sul, Brazil; 3 Department of Physiotherapy, Universidade Federal de Ciencias da Saude de Porto Alegre, Porto Alegre, State of Rio Grande do Sul, Brazil

**Keywords:** Osteoarthritis, Hydrotherapy, Protocol

## Abstract

**Background:**

Knee osteoarthritis affects the performance of daily activities, independence, and quality of life. The etiopathogenesis of this condition considers the mechanisms of activation of metalloproteinase and reactive oxygen species production pathways. Metalloproteinases-3 (MMP-3) and Glutathione Peroxidase (GPx) may be responsible for cartilage destruction. Aquatic physiotherapy promotes a positive impact on the clinical picture of osteoarthritis, and this study presents an intervention protocol that aims to evaluate the effects of a single session of different aquatic physiotherapy modalities on the biochemical and functional behavior of patients with knee osteoarthritis.

**Methods:**

This will be a crossover randomized controlled trial in which 15 individuals will be submitted to three aquatic physiotherapy modalities with a minimum 15-day wash-out period in patients over 50 years old and diagnosed with OA in at least one knee, presence of pain and at least one functional dysfunction for at least 6 months, absence of physical limitation that prevents the exercise protocol from being performed, Kellgren and Lawrence ranking between I and IV, walk independently and without auxiliary device. Variations in the concentrations of MMP-3 and GPx in peripheral blood, pain, edema, and flexibility resulting from the three aquatic physiotherapeutic interventions will be evaluated both pre- and immediate post-intervention. The reference group will be submitted to the same aquatic physiotherapy protocols, however, only the biochemical parameters and the self-perception questionnaires will be evaluated.

**Registration:**

ClinicalTrials.gov ( NCT05610696, 18/01/2023).

## Introduction

Osteoarthritis is the most prevalent rheumatologic joint dysfunction in the world, the knee joint can be considered the most affected segment and is due to local and systemic risk factors. ^1^^,^^2^ Knee osteoarthritis affects its functionality, mainly the performance of activities of daily living such as locomotion or even the transfers necessary to maintain independence and quality of life. ^3^^,^^4^ The evolution is generally slow and may evolve in stages or reflect as a progressive evolution over time, resulting in a worsening of the severity of the symptoms and disease, ^5^ which also has an important economic and social impact. ^6^ According to the data from *vizhub.healthdata.org* , Brazil is in the 15 ^th^ position in the distribution ranking of osteoarthritis cases, and a prevalence of 3.42 cases per 100,000 inhabitants is estimated, with an incidence of 181.47 new cases.

It is currently assumed that inflammatory processes osteoarthritis is strongly related to the genesis of osteoarthritis and, in this sense, the articular cartilage, the target of degenerative processes, is modified in its composition and structure. ^7^ As deterioration progresses, collagen fibers degenerate due to the secretion of collagen-degrading enzymes called metalloproteinases (MMPs), ^8^ as well as the generation of reactive oxygen species (ROS) that cause oxidation and subsequent damage to articular components. ^9^^–^^12^

Many MMPs families have been reported to induce articular cartilage degradation. ^8^^,^^13^ The metalloproteinase-3 (MMP-3) can be considered the great responsible for the destruction of cartilage resulting from the degradation of several types of collagens, ^13^ and this same enzyme was found in the plasma shortly after articular injuries, possibly due to the inflammatory activation process of the synovium, similarly, the plasma levels of MMP-3 are shown to be predictors of articular narrowing in patients with osteoarthritis. ^14^ The oxidative stress consists of a cellular response in which the intracellular synthesis of ROS results from the cellular inability to neutralize these molecules that, when in high concentrations, induce damage and even cell death. In the case of osteoarthritis, cells in several tissues produce a large amount of ROS. ^9^^,^^15^^–^^17^

Treatment strategies for osteoarthritis are described as evidence-based practices along with guidelines that indicate consensus of important organizations dedicated to the study of musculoskeletal conditions. Most study bases are focused on knee articulation, and, in this sense, the American College of Rheumatology (ACR), ^18^ The European Alliance of Associations for Rheumatism (EULAR), ^19^ and the Osteoarthritis Research Society International (OARSI) ^20^ reinforce that the practice of exercises and physiotherapy (both on the ground and in the liquid environment) are an important therapeutic arsenal for knee osteoarthritis. Although the evidence regarding the efficacy and safety of exercise is well elucidated, there is still a need to clarify the intensity levels of this activity safely. In this regard, the type of exercise and the various parameters concerning its intensity are part of the challenge for physiotherapists dedicated to rehabilitating rheumatic diseases.

It is currently well established that aquatic physiotherapy promotes a positive impact on the improvement of the clinical and functional status of patients with knee osteoarthritis, being considered an effective and safe modality once the immersion in heated water reduces overload and joint pain in addition to improving functional capacity and quality of life. ^21^ This treatment modality also promotes the improvement of functional capacity and emotional well-being. ^22^ Instituting treatment through aquatic physiotherapy in patients with knee osteoarthritis means considering that, from the immersion, interactions occur due to forces such as drag or turbulence that directly interfere in the performance of therapeutic exercises, so the prescription and execution of aquatic physiotherapy needs to consider clinical reasoning skills to elaborate treatment planes based on hydrostatic, hydrodynamic principles, and in the physiological immersion effects, in addition to individual assessment and evidence-based practices, ^23^ these factors confer upon aquatic physiotherapy in patients with knee osteoarthritis, a treatment strategy with fewer side effects when compared to other modalities. ^24^

Likewise, studies indicate that the mechanisms for this improvement are due to the interaction between the execution of the exercise with the hydrostatic and hydrodynamic principles. ^25^^–^^27^ However, it is still necessary to understand the impact of immersion and of different modalities of aquatic physiotherapy on the biochemical behavior of articular cartilage and correlate them with functional variables. It has recently been possible to understand the effects of an aquatic exercise program on the degenerative modifications in articular cartilage using an experimental animal model of knee osteoarthritis with an alteration in the expression of MMPs ^28^ and greater integrity and orientation of collagen fibers. ^29^ Results like these indicate the possible cellular and molecular mechanisms that justify the positive results from aquatic physiotherapy in knee osteoarthritis and reinforce the need for further studies in this area. In this context, the analysis of markers such as oxidative stress and MMPs constitutes a relevant topic of study.

Previous studies in a systematic review demonstrated that aquatic physiotherapy positively affects clinical, functional, and quality of life variables in individuals with osteoarthritis. Similarly, it has recently been evidenced that aquatic physiotherapy positively affects pain, physical function, knee extension muscle strength, and walking ability. ^30^^,^^31^ These studies are related to heterogeneous protocols in intensities, frequency, total treatment time, and a single type of program.

Although this intervention has been shown to be safe, there is a lack of data evidencing its effects on biochemical variables, especially with a comparative analysis between pre-and post-intervention times, making it relevant to provide equally safe physiotherapist protocols of varying intensities. Nevertheless, understanding the central mechanism responsible for functional improvement, including the impact of aquatic physiotherapy on biochemical variables and its correlation with articular collagen degradation, will undoubtedly contribute to enhancing this type of intervention as a treatment strategy for knee osteoarthritis through evidence-based practices. These factors confer upon aquatic physiotherapy in patients with knee osteoarthritis a treatment strategy with fewer side effects than other modalities.

Therefore, the protocol proposed in this study presents different modalities of intervention in aquatic physiotherapy, this being a single session, considering low, medium, and high-intensity strategies, seeking to evaluate its effects on the biochemical and functional behavior of patients with knee osteoarthritis. It aims to identify possible variations in the concentration of the MMP-3 subfamily and the oxidative stress marker Glutathione Peroxidase (GPx) in peripheral blood, assess pain, edema, and flexibility in patients with knee osteoarthritis and in response to different aquatic physiotherapy interventions. Therefore, understanding the appropriate exercise intensity, coupled with understanding its effects on biochemical mediators directly involved in the genesis of osteoarthritis, can provide confidence for the prescription and execution of therapeutic exercises under immersion in aquatic physiotherapy treatment. These strategies may increase treatment adherence, prevent symptom exacerbation, minimize adverse effects, and provide greater therapeutic safety.

## Methods

### Trial design

This is an interventional transversal study, randomized clinical trial, crossover type with three arms: low, medium, and high-intensity aquatic physiotherapy.

**Figure 1.**
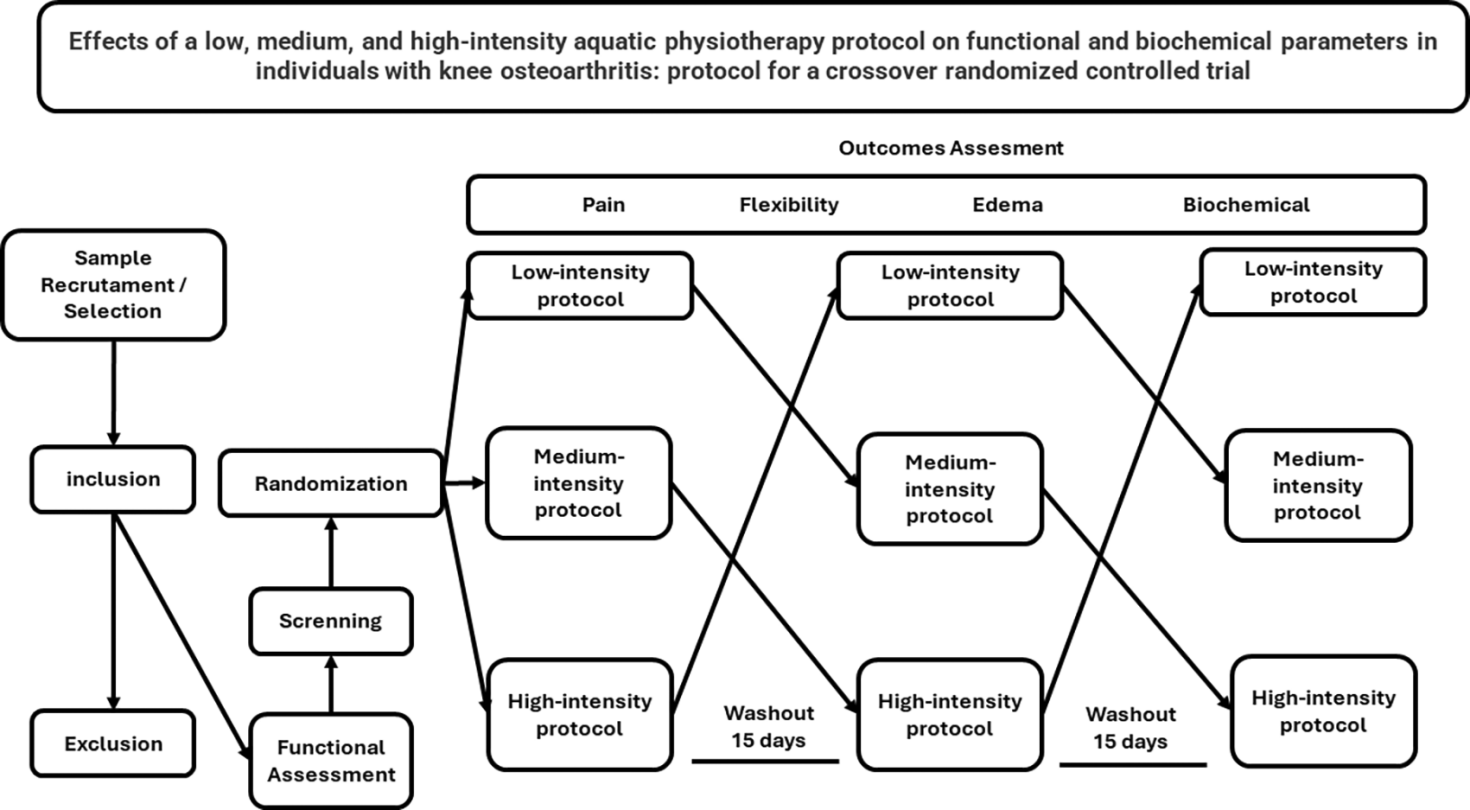
Flow of interventions and assessment parameters.

This clinical intervention protocol follows the recommendation from the Standard Protocol Items: Recommendations for Interventional Trials (SPIRIT). ^32^^,^^60^ The study sample, defined according to criteria, will be submitted to a single session of each one of the following modalities of aquatic physiotherapy: (a) low-intensity, (b) medium-intensity, and (c) high-intensity. Among each modality, a minimum of a 15-day wash-out period will be respected, in accordance with Figure 1.

For this study, 15 participants will be recruited. The sample size calculation indicated a non-centrality parameter (δ) of 3.8, suggesting a moderate effect on biomarker variations. The critical t-value was 2.10, with degrees of freedom (df) of 16.143, resulting in a total sample size of 15 participants and an actual power of 0.95. The effect size calculation was based on means and standard deviations, and for our main dependent variables, these were reported by previous investigations: Germanou ^33^ through the antioxidant levels and Nascimento ^34^ through the MMP-2 and MMP-9 analysis between the control and intervention group, in this case, it is represented by patients with knee osteoarthritis.

The assessment parameters used in each variable will consist of instruments that allow for the assessment of the variations in the MMP-3 concentration, oxidative stress, pain, edema, and flexibility. In addition, a self-perception questionnaire, developed by the authors, will be applied to assess self-perception. It consists of a Likert scale where the patient must classify their perception of well-being between zero and 10, where zero represents the worst feeling of well-being and 10 represents the best feeling of well-being. The data collection form, informed consent form and patient identification form can be found as *Extended data.* ^60^ The trial has been registered on ClinicalTrials.gov ( NCT05610696, 18/01/2023).

### Study setting

The study population corresponds to people with knee osteoarthritis. The population will be recruited from physiotherapy services from University Feevale.

The protocol will be performed in the facilities of the Aquatic Physiotherapy sector. The swimming pool has accessibility *via* stairs and a ramp with appropriate handrail support. Heating is provided by an electronic boiler fed with liquefied petroleum gas that guarantees minimum temperature variations (33°C and 34°C) measured with a submerged mercury column thermometer. The swimming pool depth varies between 100 and 150 cm, transitioned by means of a ramp; the entire swimming pool is lined with non-slip tiles. The swimming pool also has two hydromassage spaces on one of the borders. Each one is equipped with hydromassage jets, one in the trunk support region and two on the sides of this area.

### Eligibility criteria

The following eligibility criteria will be considered: being over 50 years old and diagnosed with osteoarthritis in at least one knee according to the American College of Rheumatology ^35^ criteria, referring to pain and at least one dysfunction for at least six months, absence of physical limitation that prevents the performance of the exercises protocol, have Kellgren and Lawrence classification between III and IV ^36^ and independent gait. Subjects who present autoimmune inflammatory conditions, use of psychotropic, immunosuppressant, or steroids drugs, consumption of antioxidants (vitamin E, statins, or uric acid reducers), alcohol and tobacco, progressive ankylosis states, the coexistence of neurological lesions, infections or skin diseases that contraindicate swimming pool immersion, surgical procedures on the knee joint in the last 12 months (excluding meniscectomy or arthroscopy), lower limb articular prosthesis, presence of inflammatory or infectious disease, uncontrolled systemic arterial hypertension, uncontrolled diabetes, chronic obstructive pulmonary disease and history of ischemic heart disease, self-reported aquatic phobia, and chlorine allergy will be excluded from the study. For control purposes, a reference group will be constituted with age matching and exclusion criteria identical to the experimental group.

### Outcomes

Initially, all participants will be interviewed and evaluated in relation to the degree of osteoarthritis and functionality. Information regarding the patient data will be confirmed *via* interview, considering data such as complete name, date of birth, age, affected knee(s), duration of pain, arterial blood pressure, list of medications in use, consumption of tobacco or alcohol, associated diseases, if they are undergoing physiotherapy treatment or regular physical activity.

The classification regarding the degree of osteoarthritis will be performed by analyzing the radiograph of the affected knee according to the method of Kellgren and Lawrence. The radiographs will be analyzed, the degree of osteoarthritis will be identified with the negatoscope support, and the images in the digital form will be displayed on a computer screen, considering the classification expressed in degrees ranging from zero to IV.

Subsequently, the identification of functionality will be assessed according to the Brazilian version of the Knee Injury and Osteoarthritis Outcome Score (KOOS). The KOOS questionnaire was developed in the 1990s as an instrument to evaluate the patient’s perception of their knee and associated problems. Since its first publication in 1998, the psychometric properties of KOOS were evaluated in a systemic review of 37 articles demonstrating adequate validity in its use for the study population. ^37^

Regarding the pre- and post-intervention assessment instruments, all participants will have the following parameters evaluated: concentration of MMP-3 and GPx, pain, edema, flexibility, and self-perception of the general well-being. In addition, it should be noted that in the 24-hour-follow-up, only the biochemical concentrations and the self-perception questionnaire will be reevaluated.

The biochemical variables will be measured in peripheral blood from the concentrations of MMP-3 and GPx. The levels of MMPs will be measured based on the Enzyme-Linked Immunosorbent Assay (ELISA) methodology, which is based on the antigen-antibody interaction and will be performed according to the kit manufacturer’s instructions. MMP-3, a constituent of the stromelysin family that is an important mediator responsible for the degradation of collagen type II, IX, and XI, ^13^^,^^14^ will be considered for this analysis. Oxidative stress will be measured by analyzing the peripheral blood from percutaneous punction. The sample collection time will be performed according to the study by Nascimento ^34^ and will be in accordance with the following times: baseline moment (immediately before the intervention), moment 1 (immediately after the intervention), and moment 2 (24 hours after the intervention).

The assessment of the degree of pain will be performed using the visual analogue pain scale, ^38^ from a scale graduated from zero to 10, in which zero corresponds to cases without pain and 10 corresponds to the worst imaginable pain.

The assessment of edema will be performed based on the measurement of the circumference of the affected knee(s). The measure will be performed with the patient relaxed in dorsal decubitus and the circumference will be determined with the knee in extension, based on the proximal distance of 1 cm from the base of the patella. ^39^ For that, the same measuring tape will be used for all measurements.

The flexibility will be assessed by measuring the amplitude of the active and passive movement for the knee flexion and, for that, a conventional goniometer will be used. The goniometer will be used because it has a low cost, is easy to handle, allows the assessments to be performed quickly, and the measurements and movement amplitude for the knee joint are valid and reliable. ^40^ For the assessment, the knee will be free of clothing, and the patient will be positioned in dorsal decubitus with the hip articulation at 90° of flexion. The fixed arm of the goniometer will be positioned parallel to the thigh with an orientation towards the greater trochanter of the femur and the mobile arm will be parallel to the lateral face of the fibula, directed towards the lateral malleolus. The movement reading will be performed while maintaining the final possible position of the arc of movement where the highest reading degree represents the assessed measurement. ^41^ All procedures will be performed by a single physiotherapist.

### Interventions

The subjects of this study will be submitted to different interventions of aquatic physiotherapy systematized as follows: program A (low-intensity protocol), program B (medium-intensity protocol), and program C (high-intensity protocol).

The access to the immersion will be from a ladder, and a period of 5 minutes will be allowed for acclimatization to the liquid environment. Considering that the intervention proposals aim to provide different levels of articular overload, the speed and time of execution will be gradually variable.

Program A will use the principles of Ai Chi ^®^ as this model is defined as a strategy of low intensity and consists of a sequence of slow movements. ^42^ For that, a sequence of adapted exercises ^43^ will be performed, according to Table 1.

**Table 1.** Program A: low-intensity protocol. The individuals in the images are students of the Physiotherapy course who consented and authorized their image.

Exercise	Periodization	Description
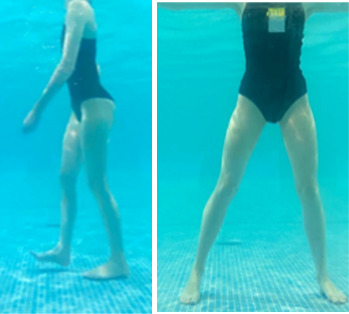	**4 minutes**	Antero-posterior and latero-lateral walk.
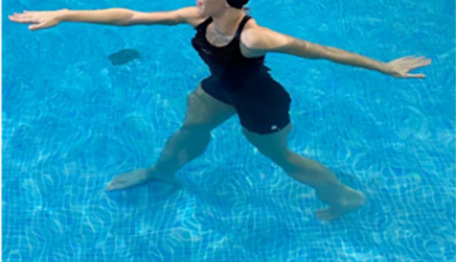	**4 minutes, 2×2 minutes**	Horizontal adduction and abduction of the shoulders alternately with rotation of the trunk to the left and right, aiming at transferring the weight in each of the lower limbs.
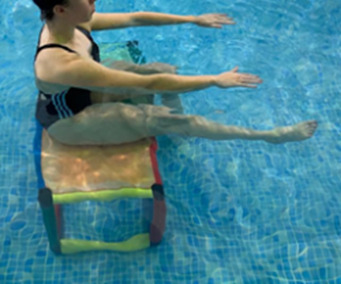	**4 minutes, 2×2 minutes**	In the sitting position, perform flexion and extension of both knees alternately.
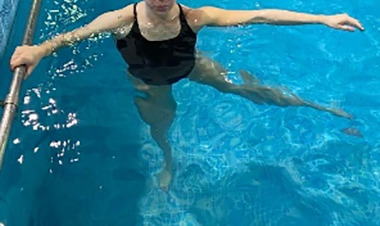	**4 minutes, 2×2 minutes for each articulation**	In orthostatism, perform hip circumduction.
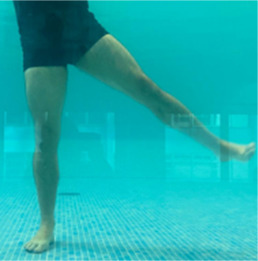	**4 minutes, 2×2 minutes for each articulation**	In orthostatism, perform abduction of both hips alternately.
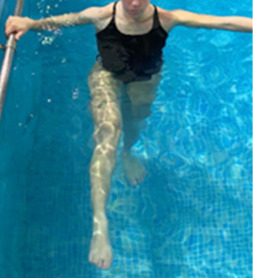	**4 minutes, 2×2 minutes for each articulation**	In orthostatism, perform flexion of both hips alternately.
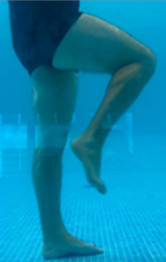	**4 minutes, 2×2 minutes for each articulation**	In orthostatism, initial position in hip flexion at 90°, perform knee flexion and extension.
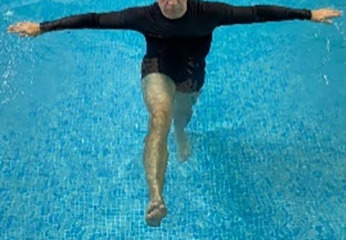	**4 minutes, 2×2 minutes for each articulation**	Elevation of the lower limb forward (single leg support) during the horizontal shoulder abduction and adduction phase. Return to bipedal support during the horizontal shoulder abduction and adduction phase.
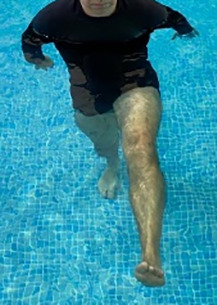	**4 minutes, 2×2 minutes for each articulation**	Hip flexion (single leg support) concomitant with shoulder extension followed by hip extension (bipedal support) concomitant with shoulder flexion.
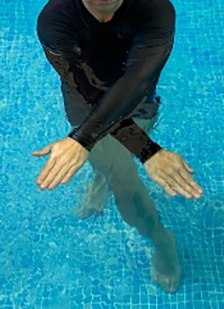	**4 minutes, 2×2 minutes for each articulation**	Cross the forearms in front of the umbilical scar, followed by the horizontal flexion together with the movement of crossing one lower limb over the other.
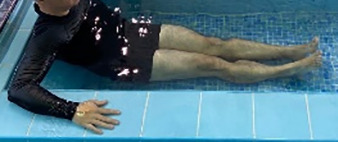	**5 minutes**	Immersion in the whirlpool bath
**Fifteen seconds rest between each exercise** **Total program time: approximately 45 minutes**		

As a starting position to perform the exercises, the participants must remain in the semi-flexion position of their knees so that the water line must remain at shoulder height throughout the procedure. All the exercises will be performed according to the established time, and a 15-second rest time will be added between each exercise or series. As a manner of manipulating the intensity of the exercise at low intensity, the movement speed will be standardized according to the subjective sensation of effort according to Borg 6-20, ^44^ with zones of interest from 6 (extremely light) to 9 (very light). In this intervention modality, we will seek to understand the impact that the physiological effects of immersion in heated water pose on the studied variables.

Program B will consist of a sequence of exercises adapted from previous works ^45^^,^^46^ composed of warm-up, fortification, and cool-down activities. Aquatic steppes platforms will be used to ensure that all participants, when performing the exercises in orthostatism, will maintain the waterline level approximately at the level of the xiphoid appendix. For this program, the exercises will be performed at a speed that guarantees a comfortable water level for the activity, being the effort zones of interest from 10 (fairly light) to 14 (somewhat hard).

The warm-up sequence consists of four different active moves performed at maximum movement amplitude and contributes to neuromuscular activation.

After the warm-up, the participants will be submitted to the fortification program, which consists of a sequence of seven exercises aiming to promote muscular activation. The participants will be encouraged to perform the movement at a comfortable speed with the maximum possible movement amplitude. During sitting exercises, the need to maintain the lumbar spine in a neutral position will be emphasized in order to avoid overloading this segment.

To finish program B, as a way to calm down, a sequence of three activities will be performed, including a period in the hydromassage area, according to Table 2.

**Table 2.** Program B: medium-intensity protocol. The individuals in the images are students of the Physiotherapy course who consented and authorized their image.

Warm-up		
Exercise	Periodization	Description
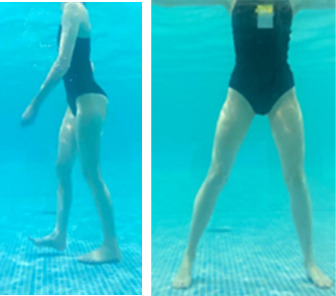	**4 minutes**	Antero-posterior and latero-lateral walk.
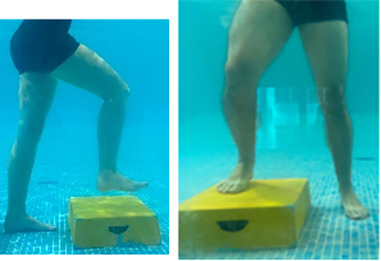	**4 minutes**	Antero-posterior and latero-lateral walk with obstacles over 30-cm-steppes.
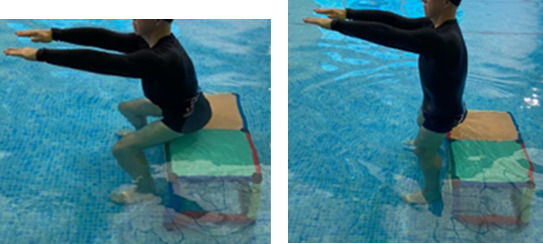	**1 minute**	Sitting and standing up.
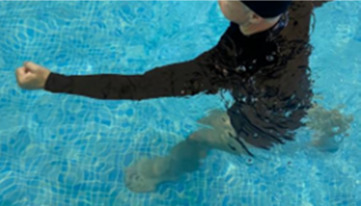	**1 minute**	Skipping - running on the same place.
**Total warm-up time: approximately 10 minutes**		
**Fortification**		
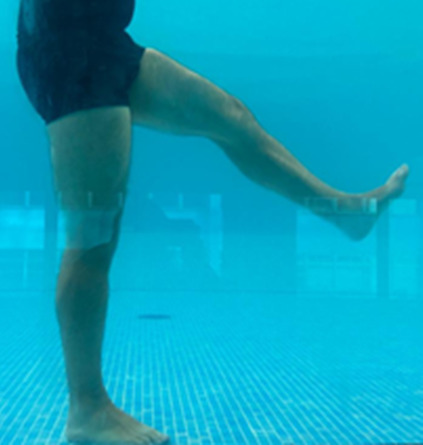	**3 minutes**	In orthostatism with unipedal support of the lower limb contralateral to the moved segment, perform hip flexion and extension. The knee must remain in complete extension and the ankle in dorsiflexion. The lumbar spine must remain in a neutral position.
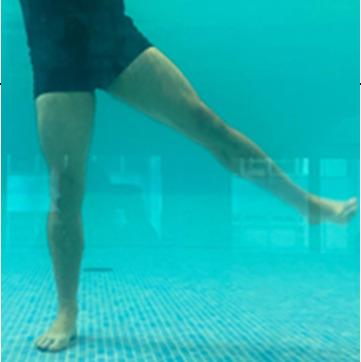	**3 minutes**	In orthostatism, perform hip abduction and adduction. The knee must remain in complete extension with the ankle in dorsiflexion. The movement must contemplate the maximum amplitude with the maintenance of the pelvis and lumbar spine in a static position.
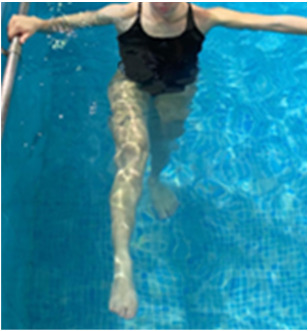	**3 minutes**	In orthostatism, perform the movement of knee flexion and extension. The movement must contemplate the maximum flexion and extension with the maintenance of the lumbar spine in a neutral position.
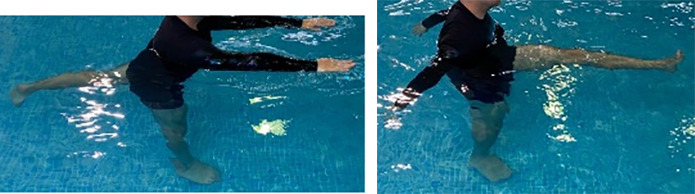	**3 minutes**	In orthostatism, perform the wide movement of hip extension and knee flexion followed by hip flexion and knee extension (kick movement).
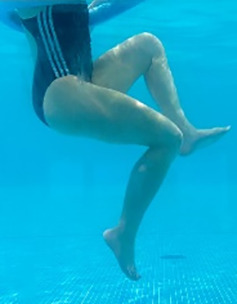	**3 minutes**	With the help of floats, perform the bicycle movement (alternating knee and hip flexion) aiming at the anterior displacement.
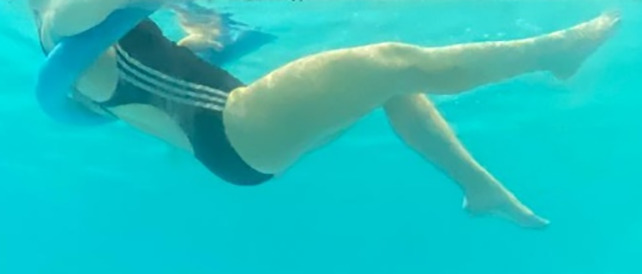	**3 minutes**	Patient in a supine position with the help of floats, perform the backstroke leg kick aiming at the cranial displacement.
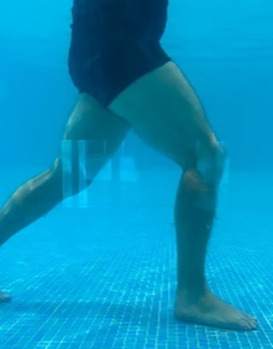	**3 minutes**	In orthostasis, perform the slow walking movement, simulating a Nordic walk, sliding the feet on the bottom of the pool, however, without moving.
**Program B fortification program time: approximately 21 minutes.**		
**Cool down**		
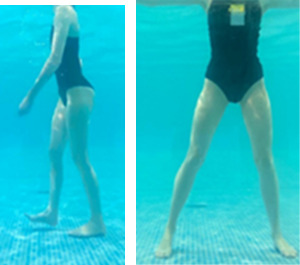	**1 minute**	Antero-posterior and latero-lateral walk.
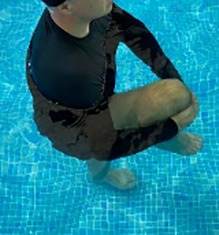	**30 seconds on each side**	Active stretching of the gluteus maximus.
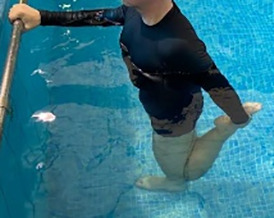	**30 seconds on each side**	Active stretching of the quadriceps.
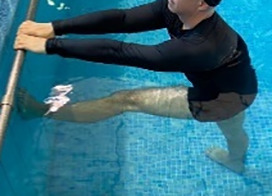	**30 seconds on each side**	Active stretching of the hamstring.
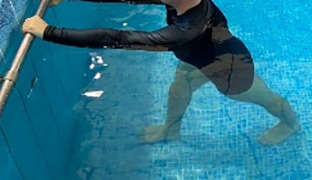	**30 seconds on each side**	Active stretching of the gastrocnemius.
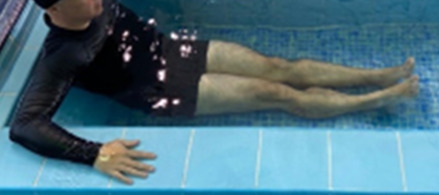	**2 minutes**	Immersion in the whirlpool.
**Total cool down time: approximately 8 minutes.**		

In program C (high-intensity protocol), the same sequence of exercises stipulated for program B will be respected, however, the quantity of work will be modified based on the variables time and speed of the performance of the movement. ^47^ Fortification activities will be performed for 6 minutes, and the speed must be adjusted in order to stimulate the performance of the movement for the subjective sensation compatible with the zone of interest 15 (hard) to 18 (very hard). In the same way, walking activity will be replaced by running activity which amounts to an approximate time of 39 minutes.

The reference group will be submitted to the same aquatic physiotherapy protocols, however, only the biochemical parameters and the self-perception questionnaires will be evaluated.

### Recruitment

The population will be recruited from the physiotherapy services of Universities. The study will also be disseminated in print and digital media, social networks of the researcher, the university, the academic directory of health courses, the Medical Leagues and institutional pages.

### Assignment of interventions

*Allocation:* Participants will be allocated randomly to establish the sequence within each group, following a computer-generated randomization schedule from randomize.com, as exemplified by the flowchart in Figure 1.

*Blinding:* This study is classified as single-blinded, indicating that the researchers responsible for conducting the assessment instruments and laboratory analysis procedures will be distinct from those administering the intervention protocols.

### Data collection, management, and analysis

*Data collection methods:* The interviews, evaluation of radiological images, application of instruments and measurements of pain, edema, and flexibility will be carried out only by a single qualified, trained physiotherapist, with familiarity with the instruments and with adequate training. Blood sample collection will be performed by a nursing professional familiar with this procedure. Laboratory analyzes will be performed by another researcher qualified biomedical pharmacist. The application of the intervention protocols will be carried out by two physiotherapists trained in aquatic physiotherapy and who will not participate in the interviews, radiological analysis and evaluation of edema, pain and flexibility.

*Data management:* All data will be entered electronically *via* Microsoft Excel. The study’s digital forms will be inserted and received in a cloud file with access only to the researcher. Participant files will be stored in numerical order and stored in storage for a period of 3 years after completion of the study. Modifications to data recorded in the database will be documented through the data breach system associated with your user identification code and password. Access to study data will be restricted. All reports will be prepared in such a way that no subject can be identified.

### Statistical analysis

The resulting data will be tabulated, considering the absolute values and their respective standards. Information regarding the main outcome corresponding to the variations in the concentrations of MMP-3 and GPx in peripheral blood due to three different aquatic physiotherapy interventions will be comparatively evaluated among the distinct interventions and among the different times (in pre- and post-intervention moments).

The information on the outcome regarding pain, edema, and flexibility will be comparatively assessed among the different types of intervention. Statistical analyses will be performed in the SPSS software and will be considered as a significance level when p>0.05 through the repeated measures ANOVA statistical test.

### Monitoring

*Data monitoring*

We perceive the establishment of a Data Monitoring Committee as unnecessary due to the brief duration of this study and the established safety levels associated with aquatic physiotherapy for patients diagnosed with knee osteoarthritis.

*Harms*

In our study, an adverse event will be defined as any unfavorable functional occurrence in an individual consistently linked to exercise intensity. Adverse events will be documented after the subject provides consent and enrollment in the study. Researchers will evaluate the connection between an event and the exercise intensity of the study protocol, based on a temporal relationship, in addition to assessing whether the event is unexpected or unexplained. The study will meticulously track the subsequent protocol-associated adverse effects: heightened pain, inflammation, and diminished flexibility immediately post-intervention and at the 24-hour mark through patient examination. For any circumstance that may necessitate urgent medical support, the emergency medical service of the Aquatic Physiotherapy department will be activated to ensure the requisite and appropriate assistance.

*Auditing*

Regarding this matter, we find it unnecessary due to the nature of our single-center study, which involves a limited sample size and employs a therapeutic approach that has been well-established as safe.

### Ethics and dissemination

*Ethics approval and consent*

This research was approved by the Research Ethics Committee of Federal University of Health Sciences in Porto Alegre in 15/05/2020 founder the number 4.030.865 and Presentation Certified for Ethical Appreciation (CAAE) number 29447220.5.0000.5345. Patients will be invited to participate in the study voluntarily, and they must consent to their participation by formalizing the Free and Informed Written Consent according to established by resolution number 466 from the National Health Council. ^48^ The study is planned to take place at University Feevale, and ethical approval has been obtained from the Research Ethics Committee of the Federal University of Health Sciences in Porto Alegre. This choice of institution for ethical approval is due to a strategic partnership between the two universities for this research endeavour. The partnership was established because our research advisor is affiliated with the Department of Physical Therapy at the Federal University of Health Sciences, making this institution the natural choice for ethical oversight. This partnership ensures that both institutions are jointly responsible for the successful execution of the study and ethical compliance is maintained throughout. The University Feevale is a co-participating institution in the research and, as such, declares its compliance with Resolution CNS 466/2012, which pertains to Brazilian regulations for research involving human subjects.

*Protocol amendments*

While the prescribed protocol has been deemed safe, having undergone pilot interventions within the aquatic physiotherapy department of the university, any modifications that might impact the study’s execution will necessitate renewed consideration by the Ethics Committee.

*Confidentiality*

Participant study data will not be disclosed beyond the study without explicit written consent from the participants. This confidentiality is upheld through the previously approved Informed Consent Form.

*Access to data*

Sole access to the datasets will be granted to the Principal Researcher. Project data shall be securely stored within password-protected files. To ensure privacy, data shared with project team members will be stripped of any participant-identifying information.

*Ancillary and post-trial care*

The responsible researcher will promptly provide comprehensive support to research participants concerning potential complications and resulting implications. Similarly, considering the research’s conduct within an accredited aquatic physiotherapy service, the service will consistently uphold any requisite therapeutic follow-ups after the study.

*Dissemination*

The study will be disseminated in printed and digital media, on the researcher, university, and the academic directory of health courses social media, in the Medicine Leagues, and on institutional pages.

**Study status**

Not yet recruiting.

## Discussion

The protocol presented in this study demonstrates different modalities of a single intervention in aquatic physiotherapy considering low, medium, and high-intensities strategies and aims to evaluate their effects on biochemical behavior in pain, edema, and flexibility in patients with knee osteoarthritis.

The proposal of low-intensity intervention consists of movements that involve the upper and lower limbs and trunk and are highly recognized by their relaxation capacity, potentiated by the interactions between their practitioners and the properties of the liquid environment. ^49^ This strategy has proven to be an effective method in the improvement of the symptoms and quality of life of patients diagnosed with Fibromyalgia, ^50^ reducing the risk of falls in older adults and producing an improvement in gait. ^51^ Regarding knee osteoarthrosis, this treatment option demonstrated to be capable of positively interfering in the reduction of pain, stiffness, functionality, and the quality of life because of the thermal effects of the immersion and the interactions provided by the physical property of water associated with slow and relaxing movements. ^43^

The aquatic physiotherapy intervention, when implemented, must take into consideration the body constitution, the speed of the movement, and the depth of the water as fundamental determinants to compose the intensity of the programmed exercise. Moving a segment in water requires more effort than moving oneself on the ground. The reduction of body weight experienced with the immersion also reduces the load on articulations affected by osteoarthritis and allows the performance of the closed-chain functional exercises that might otherwise be very difficult to perform on land. In the same way, the water turbulence might be used as a method to increase the resistance, and the percentage of body weight carried by the lower limbs can be decreased or increased directly proportional to the immersion depth. ^23^

Therefore, using the physical principles of the water becomes a great ally when aiming to treat individuals in this environment. For example, floating can reduce pain in patients with osteoarthritis since immersion is directly related to the reduction of body weight. In this situation, decreasing load means reducing articular pain, allowing a patient with osteoarthritis to perform exercises where the body weight variations can be constituted as an important therapeutic strategy for performing functional exercises, which is hard to adapt to the ground. ^25^

The hydrostatic pressure is directly related to the immersion depth, which has a positive effect on venous blood return and reduction of edema. ^27^ Water can retain the temperature and is an important thermal conductor capable of transferring thermal variations to the immersed body. The aquatic physiotherapy programs performed at temperatures ranging from 33.5-35.5°C allow heat transfer to the segments in contact with water and pain relief. ^52^

Widely used, aquatic exercises provide positive results in joint mobility and muscle strength because, with the immersion, the impulsion force provided by floating reduces the weight of body segments in a directly proportional way to the volume of displaced water, and this condition favors the movement of individuals with muscle weakness. ^53^

Benefits such as an improvement of functionality, muscle strength, and quality of life are observed in patients with osteoarthritis and other skeletal muscle disorders after aquatic physiotherapy. ^52^ The implementation of treatment programs for knee osteoarthritis can also be planned from high-intensity exercises. This option of intervention is from adjustments in variables such as the increase in the period of training (weeks, months), frequency (days, weeks), session (minutes), or the amount of effort generated with the activity (heart rate, effort). This way, the composition of a program of high-intensity *versus* low-intensity must take into consideration the performance of the same program of exercises or physical activity in both groups, however, with different intensities. ^47^

Considering that this protocol aims to explore the effects of different programs of aquatic physiotherapy, to increment the work imposed during the performance of each one of the programs, the variables time, resistance, and movement speed will be proportionally modified. High-intensity training is known to bring benefits by contributing to functional performance and reducing the risk of falls for patients with knee osteoarthritis, and when performed in an aquatic environment, producing less load than when performed on the ground. ^54^ Lower articular load and lower generation of pain are clearly observed in squat exercises performed in a swimming pool, which indicates the great therapeutic potential of aquatic physiotherapy for patients with this condition. ^55^

The swimming pool walk is an important component for the improvement of the aerobic capacity of patients with osteoarthritis. This activity in the liquid environment requires speeds 30% lower than on the ground, while accelerating walks demand an increase of work of the cardiovascular system and require more strength for its execution. Walking with water at a depth corresponding to the xiphoid appendix reduces the articular overload, however, the increase in the speed can selectively recruit the hip extensor musculature, while walking backward reduces the patellofemoral compression, relieving compressive loads on this joint. ^56^ Running in the water is also considered an important intervention strategy for patients with osteoarthritis, as it demands a varied combination of exercises involving upper and lower limbs. ^56^^–^^58^

Nevertheless, although the improvement of functional variables in patients with osteoarthritis is widely unknown, the understanding of molecular mechanisms and their effects on articular cartilage are still scarcely explored. The effects of an aquatic exercise program on degenerative modifications of articular cartilage in an experimental animal model of knee osteoarthritis evidenced alterations in MMPs concentrations ^27^ and demonstrated that aquatic physiotherapy was able to produce more integrity and orientation of collagen fibers. ^28^ In this context, the analysis of biochemical markers constitutes a relevant topic of study by evaluating the effects of different modalities of aquatic physiotherapy on the functional and biochemical behavior of patients with knee osteoarthritis and becomes more relevant when seeking understanding and answers obtained in different work intensity programs on articular cartilage. The expected results with one single session will be indicative for future longitudinal studies based on assertive intervention proposals regarding protocols with lower production of biochemical agents responsible for collagen degradation.

Finally, immersion-based physiotherapeutic treatment can prevent cartilage degeneration, inhibit inflammation, and prevent subchondral bone loss, as the presented evidence indicates that this modality can improve pain, stiffness, joint dysfunction, and muscle weakness in patients with knee osteoarthritis. Similarly, various treatment options are characterized by exercises of different intensities, such as aerobic exercises or strength training. ^59^ Thus, the expected results in this protocol may support future longitudinal studies, contributing to assertive intervention proposals and constituting protocols aimed at reducing the production of biochemical agents responsible for collagen degradation.

## Conclusions

The positive effects of aquatic physiotherapy on functional variables in patients with knee osteoarthritis are widely known and studied, however, it is still necessary to deepen the understanding of this treatment modality on the interaction of biochemical mediators from the degenerative process of articular cartilage. Likewise, understanding how different exercise intensities affect these variables is essential, since the safe modulation of exercises intensities is one of the important parameters in the treatment of aquatic physiotherapy. In this way, the study of the effects of programs of variable intensities in a single session is the object of the present protocol, it may provide subsidies for new longitudinal studies aimed at the lower production of biochemical agents of articular degradation.

The authors believe that this study, while providing important information, has some limitations. First, biomarkers considered important for understanding knee joint metabolism will be used. However, the inclusion of Cartilage Oligomeric Matrix Protein (COMP), a protein found in the extracellular matrix of cartilage, tendons, and other connective tissues, as well as other metalloproteinases and the analysis of inflammatory cytokines such as IL-1, IL-6, IL-8, and TNF-α, could offer a more detailed analysis of the acute effects of aquatic therapy on metabolism across all joint tissues. Second, the systemic response of the markers will be assessed. The authors are aware that analyzing intra-articular samples could provide information about this microenvironment; however, extracting intracavitary knee samples poses a significant challenge for implementing exercise in immersion, as sample collection involves invasive procedures in the joint cavity, which could contraindicate immersion immediately after collection. Third, interlaboratory variability and slight variations in existing study protocols can make it extremely difficult to compare results from different interventions to better understand the role of exercise characteristics and their impact on serum concentrations of soluble markers in this protocol. Fourth, the objective is to analyze the acute effects of different aquatic therapy protocols, but it is understood that future longitudinal studies, supported by the results from the implementation of this protocol, could provide better information on long-term interventions.

## Data Availability

No data are associated with this article. Harvard Dataverse: SPIRIT. https://doi.org/10.7910/DVN/FJ1RBN. ^60^ This project contains the following extended data:
- Data Collection Form.docx - ICF.docx - Patient Identification Form.docx - SPIRIT-Checklist.doc Data are available under the terms of the Creative Commons Zero “No rights reserved” data waiver (CC0 1.0 Public domain dedication).
