# Supplementary material for: Effects of a low, medium, and high-intensity aquatic physiotherapy protocol on functional and biochemical parameters in individuals with knee osteoarthritis: protocol for a crossover randomized controlled trial
Source: F1000Res. 2024 Sep 16;12:1605. Originally published 2023 Dec 29. [Version 4] doi: 10.12688/f1000research.140342.4 (PMC11409911; doi:10.12688/f1000research.140342.4)
Supplement: Peer review history

# Reviewer report: Reviewer response for version 4

**DOI:** 10.5256/f1000research.171009.r324080

**Published:** 2024-09-17

**Authors:** Domiziano Tarantino

**Affiliations:** 1 University of Naples Federico II, Naples, Italy; 2 Fondazione Don Carlo Gnocchi, Salerno, Italy

I have no further comments to make.

Is the study design appropriate for the research question?

Yes

Is the rationale for, and objectives of, the study clearly described?

Yes

Are sufficient details of the methods provided to allow replication by others?

Yes

Are the datasets clearly presented in a useable and accessible format?

Yes

Reviewer Expertise:

Physical Medicine and Rehabilitation

I confirm that I have read this submission and believe that I have an appropriate level of expertise to confirm that it is of an acceptable scientific standard.

# Reviewer report: Reviewer response for version 3

**DOI:** 10.5256/f1000research.168059.r308950

**Published:** 2024-08-08

**Authors:** Domiziano Tarantino

**Affiliations:** 1 University of Naples Federico II, Naples, Italy; 2 Fondazione Don Carlo Gnocchi, Salerno, Italy

Dear Authors,

 Even if the overall quality of your study protocol is good and with a solid scientific rationale, I am skeptical about such extremely short follow-up (24 hours). Please help me understand how the findings could be useful in clinical practice since osteoarthritis is a chronic conditions that needs therapeutic solutions in the long-term.

 Kindly check the typing mistakes throughout the entire manuscript and some sentences need to be rephrased (such as "It is currently assumed that inflammatory processes osteoarthritis is strongly related to the genesis of osteoarthritis" or "instituting treatment through aquatic physiotherapy in patients with knee osteoarthritis means considering that", etc).

 Some references are needed, such as at the end of the following sentence: "Previous studies in a systematic review demonstrated that aquatic physiotherapy positively affects clinical, functional, and quality of life variables in individuals with osteoarthritis."

**Methods**

 Figure 1 is quite confusing. I would suggest to remake it.

 No reference to what patients did or were making before the 15-day wash-out in terms of physiotherapy and/or therapeutic exercise.

**Outcomes**

 I would use T0, T1, T2 instead of moment 1 and moment 2

**Interventions**

 When talking about high-intensity, please specify that you are referring to the Borg scale.

 Limitations of the study should be moved at the end of the Discussion.

 You should add the following references:

 1) Tarantino D et al. (2023 [Ref - 1])

 2) Tarantino D et al. (2023 [Ref - 2])

Is the study design appropriate for the research question?

Yes

Is the rationale for, and objectives of, the study clearly described?

Yes

Are sufficient details of the methods provided to allow replication by others?

Yes

Are the datasets clearly presented in a useable and accessible format?

Yes

Reviewer Expertise:

Physical Medicine and Rehabilitation

I confirm that I have read this submission and believe that I have an appropriate level of expertise to confirm that it is of an acceptable scientific standard, however I have significant reservations, as outlined above.

## References

[1] : High-Intensity Training for Knee Osteoarthritis: A Narrative Review. *Sports (Basel)* .2023;11(4) : 10.3390/sports11040091 10.3390/sports11040091 37104165 PMC10141118

[2] : Intra-Articular Collagen Injections for Osteoarthritis: A Narrative Review. *Int J Environ Res Public Health* .2023;20(5) : 10.3390/ijerph20054390 10.3390/ijerph20054390 36901400 PMC10001647

# Response

**Published:** 2024-08-24

**Authors:** Gustavo de Castro

Dear Reviewer Mr. Tarantino,

 We sincerely appreciate your excellent contributions to our article. We truly feel honored to have you involved in such a critical process for our research group.

 This protocol represents the first step in a series of future studies exploring the relationship between biomarkers and different intensities of Aquatic Physiotherapy. Our aim with this protocol also includes an immediate evaluation of the safety of various protocols, particularly the high-intensity protocol. We recently implemented the protocol and obtained extremely encouraging results, which allow us to move forward with a future longitudinal study, including long-term follow-up. The references concerning physiotherapeutic treatments and other physical activities of the experimental group will certainly be considered in future analyses.

 Following your suggestion, we have revised the text, repositioned the section related to study limitations, adjusted the terminology regarding collection and analysis times (T0, T1, and T2), and included new references, including those you recommended.

 Throughout the text, we have emphasized that the different intensities of each protocol are modulated by the Borg rating of perceived exertion scale, as follows:

**Low-Intensity Protocol:** “... as a manner of manipulating the intensity of the exercise at low intensity, the movement speed will be standardized according to the subjective sensation of effort according to Borg 6-20, 44 with zones of interest from 6 (extremely light) to 9 (very light).”

**Medium-Intensity Protocol:** “...For this program, the exercises will be performed at a speed that guarantees a comfortable water level for the activity, being the effort zones of interest from 10 (fairly light) to 14 (somewhat hard).”

**High-Intensity Protocol:** “...zone of  interest 15 (hard) to 18 (very hard).”

 Thus, we believe that the relationship between the intensities and the Borg scale has already been adequately addressed.

 Finally, the composition of Figure 1 was adapted from the article "CONSORT 2010 statement: extension to randomised crossover trials" (available at http://dx.doi.org/10.1136/bmj.l4378). However, we would be grateful if you could provide any other suggestions for flowcharts should you feel that it needs to be further modified.

 Sincerely, Cesar Augusto Teixeira

# Reviewer report: Reviewer response for version 2

**DOI:** 10.5256/f1000research.163915.r265791

**Published:** 2024-06-04

**Authors:** Onu Ilie

**Affiliations:** 1 Department of Biomedical Sciences, University of Medicine and Pharmacy “Grigore T. Popa”, Iasi, Romania

”Effects of a low, medium, and high-intensity aquatic physiotherapy protocol on functional and biochemical parameters in individuals with knee osteoarthritis: protocol for a crossover randomized controlled trial”

 It is a well-done scientific study but whether it can be indexed or not is conditional on the clarification of some issues:

 1. 15 patients are too few to validate a study

 2. GIFs in tables cannot be viewed

 3. the study does not include limitations

 4. To be replicated the study should have patients who fall into one KL classification (e.g. KL3 or KL 4).

 5. a control group should be included in the study

 After clarification of the issues mentioned above, the article can be indexed.

Is the study design appropriate for the research question?

Yes

Is the rationale for, and objectives of, the study clearly described?

Yes

Are sufficient details of the methods provided to allow replication by others?

Partly

Are the datasets clearly presented in a useable and accessible format?

Partly

Reviewer Expertise:

Doctor of Physical TherapyUniversity LecturerHead of the physiotherapy department at the Medlife - Micromedica, Piatra Neamţ, Romania. MDT McKenzie therapistInstitution and departmentUniversitatea de Medicina si Farmacie Grigore T. Popa IasiDepartment of Biomedical SciencesSkillsExercise Science · Rehabilitation · Musculoskeletal Disorders

I confirm that I have read this submission and believe that I have an appropriate level of expertise to confirm that it is of an acceptable scientific standard, however I have significant reservations, as outlined above.

# Response

**Published:** 2024-06-17

**Authors:** Gustavo de Castro

Dear Reviewer,

 We appreciate the feedback and would like to address our considerations based on the reviewed items as follows:

1. **15 patients are too few to validate a study:** To investigate the necessary number of participants and estimate the immediate effects of exercise in different Aquatic Physiotherapy programs on the concentration levels of the joint biomarkers MMP-3 and oxidative stress, we performed a sample size calculation using GPower software, version 3.1.9.7. The input parameters included a one-tailed test, an estimated effect size of 1.0, a significance level of 0.05, and a statistical power of 0.95. This calculation assumed a normal distribution, aiming to detect differences between groups and repeated measures (within-subject effect). Previous studies, such as those by Germanou (2013) and Nascimento (2016), were used to estimate the parameters. Germanou demonstrated variation in oxidative stress, and Nascimento observed significant variation in MMP-2 and MMP-9. The GPower results were as follows: the non-centrality parameter (δ) was 3.8, indicating a moderate effect on biomarker variations, the critical t-value was 2.10, the degrees of freedom (df) were 16.143, resulting in a total sample size of 15 participants and an actual power of 0.95. These results demonstrate that the effects of the markers used in the referenced articles show a moderate response to exercise, with sufficient homogeneity for a sample size of 15 individuals. We have included this information in the third paragraph of the Trial Design section.
2. **GIFs in tables cannot be viewed:** The figures in all tables are static and, according to our access, are available on the website where the article is published ( https://f1000research.com/articles/12-1605/v2#referee-response-265791). They are also visible in the PDF version. We will report your observation to the publisher, who should find a solution to make them viewable directly on the website. This adjustment depends on the publisher and not the authors.
3. **The study does not include limitations:** We appreciate the suggestion, and the limitations have been included in the text.
4. **To be replicated, the study should have patients who fall into one KL classification (e.g., KL3 or KL4):** We have addressed this suggestion, and the change has been included in the new version of the article.
5. **A control group should be included in the study:** We confirm that we have a control group. The last sentence of the Eligibility Criteria section states: "For control purposes, a reference group will be constituted with age matching and exclusion criteria identical to the experimental group."

Sincerely,

 Cesar Augusto Teixeira

# Reviewer report: Reviewer response for version 1

**DOI:** 10.5256/f1000research.153684.r246658

**Published:** 2024-03-06

**Authors:** Qian Zhao

**Affiliations:** 1 Shanxi Provincial People's Hospital, Shanxi, China

The submitted research delves into the effects of varying intensities of aquatic physiotherapy regimens on the functionality and biochemical parameters of patients with knee osteoarthritis, a topic of high significance. I commend the authors for initiating such a valuable endeavor.

 However, in my opinion, the authors should commence their introduction or theoretical framework by addressing the initial challenges present in the treatment of rheumatic joint disorders. They should then broaden the discussion to encompass the primary objectives of their research in osteoarthritis, including the authors' eventual determination of the importance and necessity of low, medium, and high-intensity aquatic physiotherapy. They should also reiterate commendation for this initiative, which has been discussed in recent years in relation to other rheumatic joint functional disorders and inflammatory diseases.

 Another key point to highlight is that in terms of the study design, Figure 1 appears to lack the representation of a crossover design trial. Given the study's low, medium, and high-intensity aquatic physiotherapy combinations, there are six different sequences of administration, which are not apparent in the figure.

 Moreover, how was the final sample size of 15 determined? The paper seems to lack clarification on this matter. I suggest that the authors present the process of determining the required sample size, which would enhance the paper's content.

 Following the aforementioned recommendations, the discussion of the obtained data should be more comprehensive.

Is the study design appropriate for the research question?

Partly

Is the rationale for, and objectives of, the study clearly described?

Partly

Are sufficient details of the methods provided to allow replication by others?

Yes

Are the datasets clearly presented in a useable and accessible format?

Yes

Reviewer Expertise:

Rheumatism immunology

I confirm that I have read this submission and believe that I have an appropriate level of expertise to confirm that it is of an acceptable scientific standard, however I have significant reservations, as outlined above.

# Response

**Published:** 2024-03-15

**Authors:** Gustavo de Castro

We appreciate your pointed considerations, which undoubtedly contributed to the refinement of the study. Given the high global prevalence of this pathology, we understand the significance of this study. Similarly, we uphold our intervention strategy within a safe physiotherapeutic approach represented by Aquatic Physiotherapy. Therefore, we aim to investigate the relationship between the behavior of two biochemical variables involved in the pathogenesis of knee osteoarthritis in Aquatic Physiotherapy programs of varying intensities. We hope our findings may eventually contribute to an exercise prescription with reduced production of biochemical agents responsible for collagen degradation. We acknowledge this as a significant challenge for physiotherapists dedicated to knee osteoarthritis treatment using Aquatic Physiotherapy. Based on your contributions, we have sought to reinforce this issue in the Introduction and discussion sections.

 Regarding the sample size, we relied on two previous studies that analyzed the biochemical variables under study. These studies were conducted on individuals with knee osteoarthritis undergoing exercise. This consideration is stated in the article's Methods section. To minimize any potential doubts, we clarify the essence of the previous studies in the text, specifically concerning the profile of the subjects investigated.

 Finally, as per your suggestion, we propose a new layout for Figure 1.
